# Advanced Versus Invasive Breast Cancer Risk in a Screening Population: Implications for Risk-based Prevention and Screening Strategies

**DOI:** 10.1007/s11606-026-10237-5

**Published:** 2026-02-18

**Authors:** Brian L. Sprague, Charlotte C. Gard, Shuai Chen, Jeffrey A. Tice, Anna N. A. Tosteson, Hannah Perry, Christoph I. Lee, Diana L. Miglioretti, Karla Kerlikowske

**Affiliations:** 1 Office of Health Promotion Research, Department of Surgery, University of Vermont Larner College of Medicine, Burlington, VT USA; 2 Department of Radiology, University of Vermont Larner College of Medicine, Burlington, VT USA; 3 University of Vermont Cancer Center, University of Vermont Larner College of Medicine, Burlington, VT USA; 4 Department of Economics, Applied Statistics, and International Business, New Mexico State University, Las Cruces, NM USA; 5 Division of Biostatistics, Department of Public Health Sciences, University of California, Davis, Davis, CA USA; 6 Division of General Internal Medicine, Department of Medicine, University of California, San Francisco, San Francisco, CA USA; 7 Dartmouth Institute for Health Policy and Clinical Practice, Geisel School of Medicine at Dartmouth, Lebanon, NH USA; 8 Department of Radiology, School of Medicine and Public Health, University of Wisconsin-Madison, Madison, WI USA; 9 Kaiser Permanente Washington Health Research Institute, Kaiser Permanente WA, Seattle, WA USA; 10 Department of Medicine, University of California, San Francisco, San Francisco, CA USA; 11 Department of Epidemiology and Biostatistics, University of California, San Francisco, San Francisco, CA USA; 12 General Internal Medicine Section, Department of Veterans Affairs, University of California, San Francisco, San Francisco, CA USA

**Keywords:** breast cancer, risk assessment, cancer epidemiology, cancer screening

## Abstract

**Background:**

Most risk-based breast cancer prevention and screening strategies rely on estimated invasive breast cancer risk. A recently developed model from the Breast Cancer Surveillance Consortium (BCSC) identifies women at elevated advanced cancer risk (prognostic pathologic stage II or greater) despite undergoing regular mammography screening.

**Objective:**

To describe the characteristics of women identified for risk-based interventions based on advanced versus invasive breast cancer risk.

**Design:**

Cross-sectional study.

**Participants:**

Women aged 40–74 years undergoing mammography screening in routine clinical practice at 165 radiology facilities participating in the BCSC.

**Main Measures:**

Established BCSC risk models were used to calculate cumulative 6-year advanced breast cancer risk and BCSC 5-year invasive breast cancer risk, stratified by age, race and ethnicity, breast density, and body mass index.

**Key Results:**

Among 756,971 included women, the Spearman correlation between advanced and invasive cancer risk scores was 0.75. 31.9% of women had intermediate/high (≥ 0.38%) advanced cancer risk and 32.5% had intermediate/high (≥ 1.67%) invasive cancer risk. 21.1% had intermediate/high risk in both models and 22.1% had intermediate/high risk in only one model. Thirty-five percent of women with intermediate/high invasive cancer risk had low/average advanced cancer risk, and 16% of women with low/average invasive cancer risk had intermediate/high advanced cancer risk. Among women with intermediate/high advanced cancer risk, 17.5% were age 40–49, 25.7% were Black, 69.7% had dense breasts, and 81.1% were overweight/obese. Among women with intermediate/high invasive breast cancer risk, 5.2% were age 40–49, 11.9% were Black, 56.5% had dense breasts, and 70.6% were overweight/obese.

**Conclusions:**

There are substantial differences in the characteristics of women identified as having elevated risk based on the advanced versus invasive breast cancer risk models. The advanced cancer risk model is important for identifying women who are young, Black, overweight/obese, or have dense breasts at intermediate/higher advanced cancer risk for consideration of risk-based interventions.

**Supplementary Information:**

The online version contains supplementary material available at https://doi.org/10.1007/s11606-026-10237-5.

## INTRODUCTION

There is widespread interest in developing risk-based breast cancer prevention and screening strategies that could improve effectiveness relative to current population guidelines by maximizing the potential benefits and minimizing the potential harms of interventions.^1–7^ Many established breast cancer risk models predict 5-year risk of developing invasive breast cancer,^8,9^ including the Breast Cancer Surveillance Consortium (BCSC) invasive breast cancer risk model,^10,11^ the Breast Cancer Risk Assessment Tool,^12,13^ and the Tyrer-Cuzick model.^14^ These risk models can be used to inform decision-making around chemoprevention,^15^ mammography screening start age,^16^ screening interval^7,17^, and supplemental screening.^18–20^

However, existing risk prediction models for invasive breast cancer are most accurate for estrogen receptor (ER)/progesterone receptor (PR) positive cancers,^8^ which have very high survival rates when detected at an early stage.^21^ In contrast, advanced-stage breast cancer is strongly associated with breast cancer mortality, particularly when classified using prognostic pathologic staging, which takes into account tumor grade and ER/PR/human epidermal growth factor receptor 2 (HER2) status.^22^ Interventions that prevent advanced breast cancer may reduce breast cancer mortality.

The BCSC recently developed the first risk prediction model for advanced breast cancer, which estimates a woman’s cumulative 6-year risk of developing prognostic stage II or greater breast cancer with an annual or biennial mammography screening regimen.^23^ The BCSC advanced cancer risk model was designed to identify women at risk of poor outcomes despite biennial mammography screening, such that more frequent screening or supplemental screening with MRI or ultrasound could be considered.

In this study, we characterized the distribution of BCSC advanced and invasive breast cancer risk in a large mammography screening population according to woman-level characteristics. We evaluated the correlation between the two risk model estimates, assessed cross-classification by categorical risk groups, and described variation in risk levels by key factors relevant to breast cancer prevention and screening strategies.

## METHODS

### Study Setting and Data Sources

This study used observational clinical data from 165 healthcare facilities participating in six BCSC breast imaging registries: Carolina Mammography Registry, Kaiser Permanente Washington Registry, Metro Chicago Breast Cancer Registry, New Hampshire Mammography Network, San Francisco Mammography Registry, and Vermont Breast Cancer Surveillance System.^24^ The catchment population for these registries spans urban, suburban, and rural practices and is broadly representative of racial and ethnic diversity in the USA.^25,26^ Women enter the BCSC registries when they receive a breast imaging exam at a participating healthcare facility. The breast imaging facility provides detailed demographic and breast cancer risk factor data for each woman undergoing breast imaging, along with information on imaging assessments and breast density. The BCSC registries also collect breast pathology data from radiology and/or pathology facilities in their catchment areas, and link to statewide or regional cancer registries. Each BCSC registry and the Statistical Coordinating Center (SCC) received institutional review board approval with active or passive consenting processes or a waiver of consent.

### Participants

**Figure 1:**
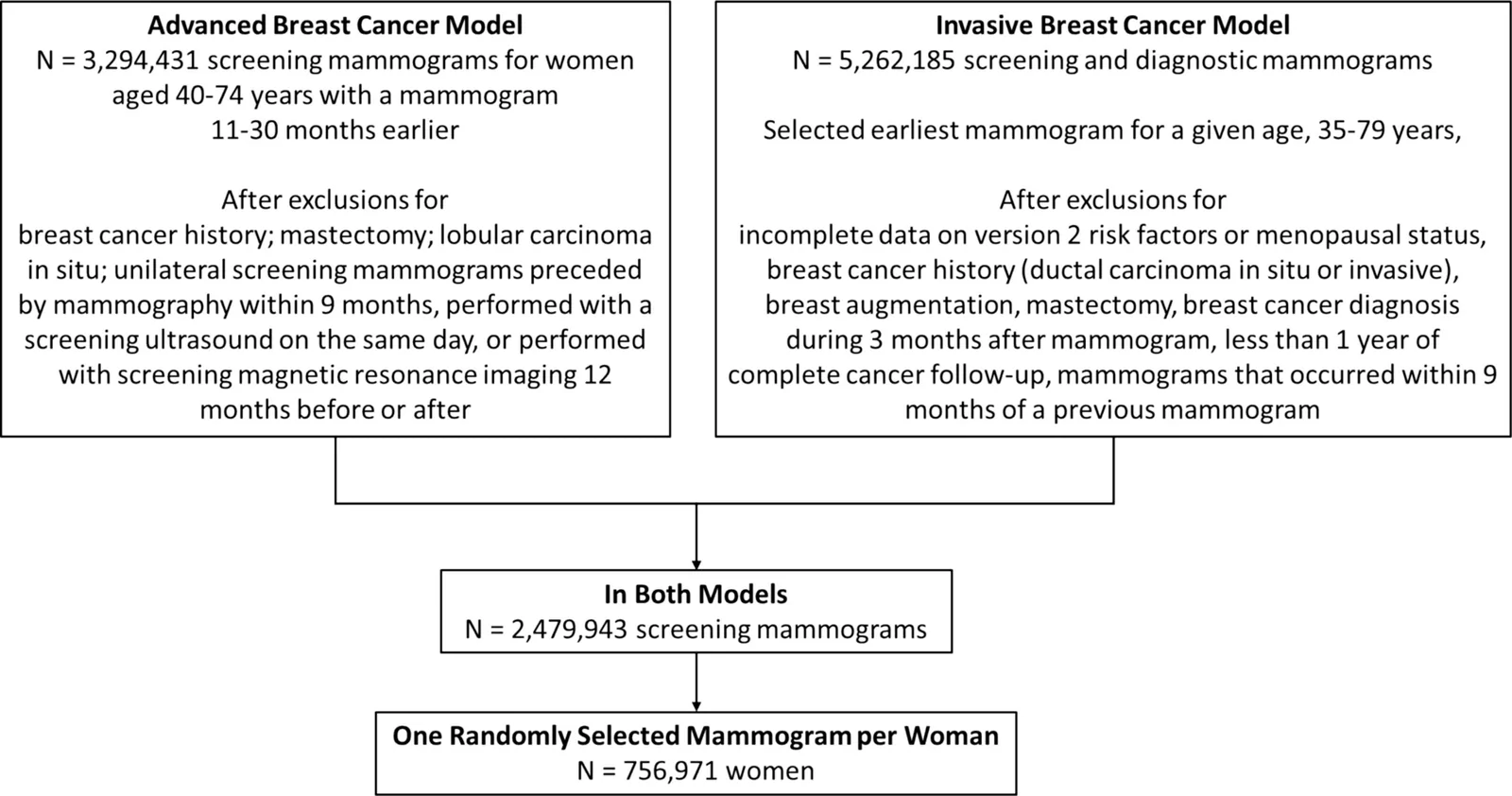
Flow diagram of inclusion and exclusion criteria for the study, which used data from previously published studies describing the development of the BCSC advanced cancer risk model^23^and the BCSC invasive cancer risk model.^10^

Participants were drawn from datasets used in previously published studies describing the development of the BCSC advanced cancer risk model and the BCSC invasive cancer risk model.^10,23^ For this study, we selected one random mammogram record per woman among the mammograms appearing in both datasets (Fig. 1). Briefly, the eligible population included women aged 40–74 who underwent an annual or biennial mammogram at a BCSC facility during January 2005 through December 2017. Mammograms among women with breast cancer diagnosed during the 3 months after the mammogram or a prior history of breast cancer, lobular carcinoma in situ, mastectomy, or breast augmentation were excluded. Mammography exams with missing data on any of the following variables required to compute BCSC invasive breast cancer risk were also excluded: age, race and ethnicity, first-degree family history of breast cancer, breast density, prior benign breast biopsy diagnosis information, and menopausal status.

### Measures, Definitions, and Outcomes

Demographic, risk factor, and clinical history data were collected from self-administered surveys at breast imaging exams and/or extracted from electronic health records. Self-reported race and ethnicity are included in the BCSC risk models because they represent a social construct that is associated with breast cancer incidence and mortality. Race and ethnicity were recorded and categorized as Hispanic or non-Hispanic; non-Hispanic women were further categorized as Asian, Black, White, or other/multiple races. Breast density was recorded by the radiologist interpreting the mammogram in routine clinical practice using the standard Breast Imaging–Reporting and Data System categories: almost entirely fatty, scattered fibroglandular densities, heterogeneously dense, or extremely dense.^27^ Women with dense breasts were defined as those with heterogeneously or extremely dense breasts.^28^ Prior benign diagnoses were categorized based on the following hierarchy in order to select the most severe diagnosis: proliferative with atypia > proliferative without atypia > nonproliferative.^29^

The woman’s BCSC 6-year advanced cancer risk^23^ and BCSC 5-year invasive breast cancer risk (version 3)^10^were computed at each mammogram. The BCSC cumulative 6-year advanced cancer risk model is based on age, race and ethnicity, first-degree family history of breast cancer, breast density, history of benign breast disease, body mass index, and menopausal status, with risk calculated based on the woman’s anticipated mammography screening interval (annual or biennial) over the next 6 years.^23^ We calculated the cumulative 6-year advanced cancer risk based on a biennial mammography screening interval, consistent with current United States Preventive Services Task Force recommendations.^30^ Women’s 6-year advanced cancer risk was classified according to previously defined thresholds as low/average (< 0.3797%), intermediate (0.3797–0.6582%), or high (> 0.6582%) risk groups.^23^

The BCSC 5-year invasive breast cancer risk model includes age, race and ethnicity, extended family history of breast cancer, breast density, history of benign breast disease, body mass index, age at first live birth, and menopausal status. Women’s 5-year invasive breast cancer risk was categorized as low/average (< 1.67%), intermediate (1.67–2.99%), or high (≥ 3.00%), consistent with previously developed thresholds.^10,29^

### Statistical Analysis

Descriptive statistics were used to describe the distribution of demographic and risk characteristics of participants. As described previously,^10,23^ multiple imputation was used to compute risk scores when risk factor data were missing. We evaluated the correlation between BCSC 6-year advanced cancer risk and BCSC 5-year invasive cancer risk using the Spearman correlation coefficient and cross-classification tables. We determined average BCSC 6-year advanced cancer risk and average BCSC 5-year invasive cancer risk by individual year of age and race and ethnicity grouping. We also determined the frequency of low/average, intermediate, and high-risk classifications for advanced and invasive cancer according to five-year age groups, race and ethnicity groups, breast density, and body mass index groups.

We computed the proportion of women identified as intermediate/high risk by age, race and ethnicity, breast density, and body mass index for each risk model. We applied these proportions to a hypothetical set of 100,000 women to tabulate the number of women who would be identified as having (1) intermediate/high 6-year advanced cancer risk; (2) intermediate/high 5-year invasive breast cancer risk; (3) dense breasts; and (4) combinations of breast density and intermediate/high advanced or invasive cancer risk. This set of calculations was repeated to characterize women with ≥ high risk.

All results were standardized to the 2020 US population based on age and race. All analyses were performed using SAS version 9.4 (SAS Institute, Cary, NC).

## RESULTS

From the original source population of 931,186 women, a total of 756,971 women met the eligibility criteria for this study (Fig. 1). The mean age of study participants was 56.1 years (standard deviation, 9.6 years) and 29.2% reported Hispanic ethnicity or non-White race (Table 1). The distribution of 6-year advanced cancer risk consisted of 68.1% low/average, 25.3% intermediate, and 6.6% high risk. The distribution of 5-year invasive breast cancer risk consisted of 67.5% low/average, 27.8% intermediate, and 4.7% high risk.

**Table 1:** Demographic and Risk Characteristics of 756,971 Women Undergoing Screening Mammography at 165 Radiology Facilities Participating in the Breast Cancer Surveillance Consortium Registries, 2005–2017

Characteristic	No.	%*
Age, years		
40–49	223,887	29.6
50–59	245,400	32.4
60–69	206,015	27.2
70–74	81,669	10.8
Race and ethnicity		
Asian	70,300	9.3
Black	91,034	12.0
Hispanic	43,872	5.8
White	535,849	70.8
Other/multiple races	15,916	2.1
Breast density		
Almost entirely fat	69,425	9.2
Scattered fibroglandular densities	326,485	43.1
Heterogeneously dense	298,311	39.4
Extremely dense	62,750	8.3
Body mass index (kg/m^2^)		
Underweight (< 18.5)	7607	1.5
Normal (18.5–24.9)	200,613	40.1
Overweight (25.0–29.9)	146,265	29.2
Obese I (30.0–34.9)	81,299	16.2
Obese II (≥ 35)	64,709	12.9
Missing	256,478	(33.9)
BCSC 6-year risk of advanced breast cancer^†^		
< 0.38% (low/average)	515,211	68.1
0.38–0.66% (intermediate)	191,588	25.3
> 0.66% (high)	50,172	6.6
BCSC 5-year risk of invasive breast cancer		
< 1.67% (low/average)	511,267	67.5
1.67 to < 3.00% (intermediate)	210,255	27.8
≥ 3.00% (high)	35,449	4.7

*BCSC*, Breast Cancer Surveillance Consortium

*Percentages given are among non-missing; counts of missing values are provided in a separate row where applicable

^†^Advanced cancer risk is calculated based on a biennial mammography regimen

The Spearman correlation coefficient between the two risk models was 0.75. Cross-classification revealed 71.7% agreement between low/average, intermediate, and high-risk categories across the two models (Table 2). 31.9% had intermediate/high (≥ 0.38%) advanced cancer risk, and 32.5% of women had intermediate/high (≥ 1.67%) 5-year invasive cancer risk. 21.1% had intermediate/high risk in both models; 22.1% had intermediate/high risk from one model but not the other; and 56.7% had low/average risk in both models. Thirty-five percent of women with intermediate/high invasive cancer risk had low/average advanced cancer risk, and 16% of women with low/average invasive cancer risk had intermediate/high advanced cancer risk. Risk categorization percent agreement was higher (77.1%) among women with non-dense breasts than among women with dense breasts (65.9%) (eTable 1 and eTable 2). Examples of advanced and invasive cancer risk score calculations are shown in Table 3, which illustrates particular differences in risk categorization among young women, Black women, and Hispanic women.

**Table 2:** Cross-classification of BCSC 6-year Advanced Breast Cancer Risk and BCSC 5-year Invasive Breast Cancer Risk

	BCSC 6-year advanced breast cancer risk*			
BCSC 5-year invasive breast cancer risk	Low/Average	Intermediate	High	
	(< 0.38%)	(0.38–0.66%)	(> 0.66%)	Total
Low/Average (< 1.67%)	429,532 (56.7%)	77,760 (10.3%)	3,975 (0.5%)	511,267 (67.5%)
Intermediate (1.67—< 3.00%)	83,512 (11.0%)	97,109 (12.8%)	29,634 (3.9%)	210,255 (27.8%)
High (≥ 3.00%)	2,167 (0.3%)	16,719 (2.2%)	16,563 (2.2%)	35,449 (4.7%)
Total	515211 (68.1%)	191588 (25.3%)	50,172 (6.6%)	756,971 (100.0%)

BCSC, Breast Cancer Surveillance Consortium

^*^Advanced cancer risk is calculated based on a biennial mammography regimen

**Table 3:** Examples of Advanced and Invasive Breast Cancer Risk Calculations for Women with Different Combinations of Age, Race and Ethnicity, Breast Density, and Body Mass Index, with No Other Risk Factors*

Age	Race and ethnicity	Breast density	Body mass index	BCSC 6-year cumulative advanced cancer risk^†^	BCSC 5-year invasive cancer risk
45	Black	Extremely dense	Overweight	0.59% (intermediate)	1.12% (low/average)
45	Hispanic	Extremely dense	Overweight	0.49% (intermediate)	0.86% (low/average)
45	White	Extremely dense	Overweight	0.50% (intermediate)	1.11% (low/average)
45	Black	Heterogeneously dense	Obese I	0.43% (intermediate)	1.78% (intermediate)
45	Hispanic	Heterogeneously dense	Obese I	0.34% (low/average)	1.47% (low/average)
45	White	Heterogeneously dense	Obese I	0.34% (low/average)	1.82% (intermediate)
55	Black	Extremely dense	Overweight	0.77% (high)	1.78% (intermediate)
55	Hispanic	Extremely dense	Overweight	0.46% (intermediate)	1.48% (low/average)
55	White	Extremely dense	Overweight	0.50% (intermediate)	1.82% (intermediate)
55	Black	Heterogeneously dense	Obese I	0.75% (high)	1.78% (intermediate)
55	Hispanic	Heterogeneously dense	Obese I	0.44% (intermediate)	1.47% (low/average)
55	White	Heterogeneously dense	Obese I	0.49% (intermediate)	1.82% (intermediate)
65	Black	Extremely dense	Overweight	0.98% (high)	2.17% (intermediate)
65	Hispanic	Extremely dense	Overweight	0.56% (intermediate)	1.88% (intermediate)
65	White	Extremely dense	Overweight	0.64% (intermediate)	2.44% (intermediate)
65	Black	Heterogeneously dense	Obese I	0.96% (high)	2.30% (intermediate)
65	Hispanic	Heterogeneously dense	Obese I	0.54% (intermediate)	1.99% (intermediate)
65	White	Heterogeneously dense	Obese I	0.63% (intermediate)	2.58% (intermediate)

*BCSC*, Breast Cancer Surveillance Consortium

*All examples assume no family history of breast cancer, no prior breast biopsies, first live birth at age 20–24, and premenopausal status for women aged 45 and postmenopausal status for women aged 55 and 65

^†^Advanced cancer risk is calculated for a biennial mammography screening regimen

**Figure 2:**
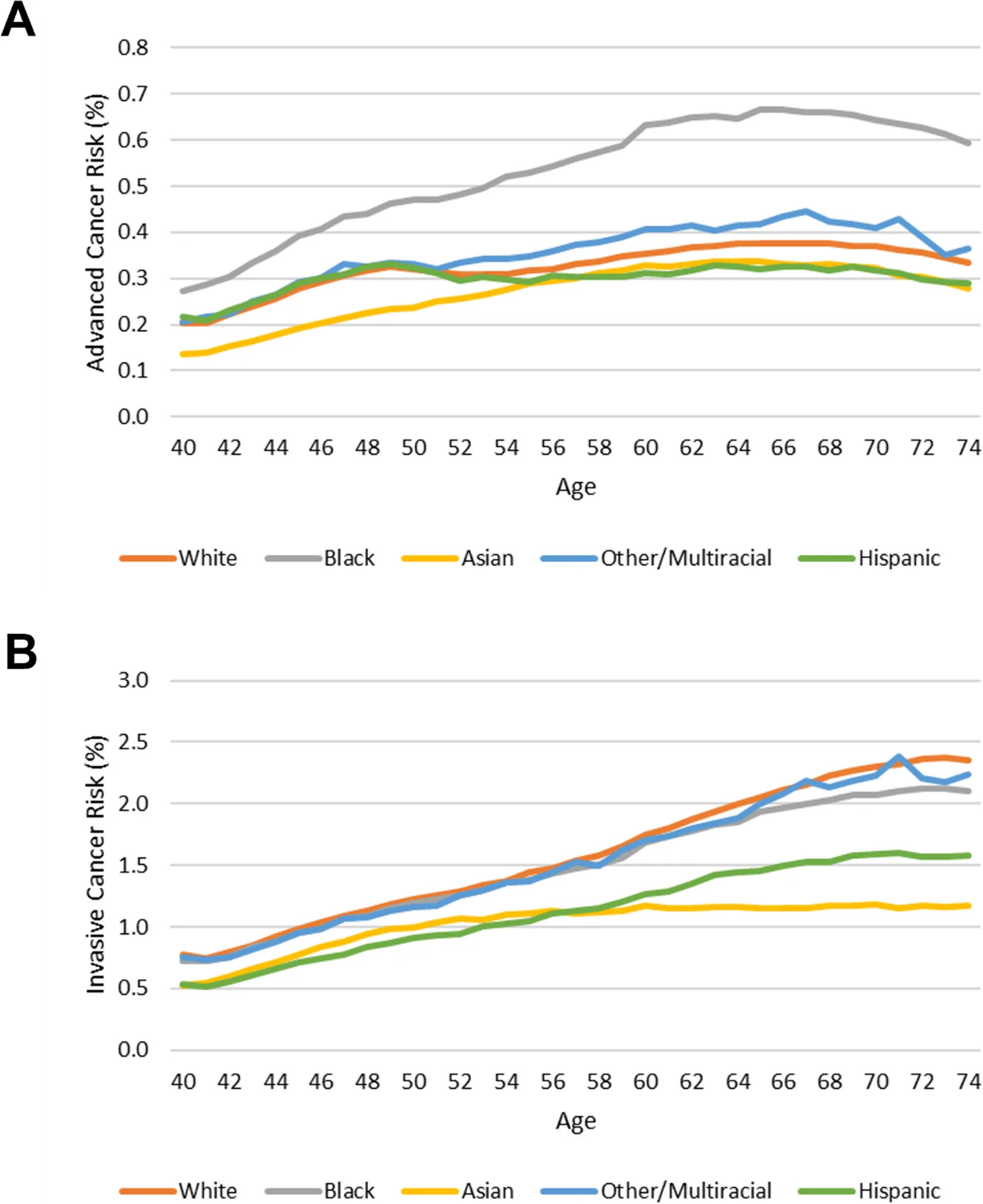
Average BCSC 6-year advanced cancer risk (**A**) and BCSC 5-year invasive breast cancer risk (**B**) by age and race/ethnicity. Advanced cancer risk is calculated based on a biennial screening regimen.

Mean BCSC 6-year advanced cancer risk plateaued near age 64 for all racial and ethnic groups (Fig. 2A). Black women had notably higher mean advanced cancer risk compared to other racial and ethnic groups. The age-standardized proportion of women with intermediate/high advanced cancer risk was 67.1% for Black women, 34.6% for other/multiple races, 29.2% for White women, 23.5% for Hispanic women, and 14.5% for Asian women (Table 4). The proportion of women with intermediate/high advanced breast cancer risk was higher among women with dense vs. non-dense breasts and among overweight and obese women compared to normal or underweight women.

**Table 4:** BCSC Advanced and Invasive Cancer Risk Classifications By Age, Race and Ethnicity, Breast Density, and Body Mass Index. Standardized to the US Population Based on age and Race

	BCSC 6-year risk of advanced breast cancer*			BCSC 5-year risk of invasive breast cancer		
	< 0.38% (low/average)	0.38–0.66% (intermediate)	> 0.66% (high)	< 1.67% (low/average)	1.67—< 3.00% (intermediate)	≥ 3.00% (high)
	Row Percentages			Row Percentages		
Age, years						
40–44	89.9%	9.3%	0.8%	97.2%	2.7%	0.1%
45–49	71.3%	24.2%	4.5%	91.1%	8.4%	0.5%
50–54	69.5%	24.8%	5.8%	83.0%	15.8%	1.2%
55–59	63.3%	29.6%	7.1%	71.7%	25.5%	2.8%
60–64	59.6%	30.2%	10.2%	53.6%	40.2%	6.2%
65–69	57.0%	33.0%	10.0%	35.8%	53.2%	11.0%
70–74	62.2%	28.7%	9.1%	24.6%	60.4%	15.0%
Race and Ethnicity						
Asian	85.5%	13.3%	1.2%	93.5%	6.2%	0.3%
Black	33.0%	42.5%	24.6%	68.3%	28.6%	3.1%
Hispanic	76.5%	20.2%	3.3%	88.6%	10.7%	0.7%
White	70.8%	24.6%	4.6%	59.6%	33.9%	6.5%
Other/Multiple races	65.4%	28.4%	6.2%	69.0%	26.5%	4.5%
Breast Density						
Almost entirely fat	99.8%	0.2%	0.0%	91.0%	8.8%	0.3%
Scattered fibroglandular densities	77.8%	19.8%	2.4%	69.4%	27.6%	3.0%
Heterogeneously dense	51.8%	36.1%	12.1%	59.5%	32.8%	7.7%
Extremely dense	49.5%	38.1%	12.4%	61.8%	32.0%	6.2%
Body Mass Index						
Underweight	69.0%	26.2%	4.8%	85.1%	14.4%	0.5%
Normal	85.1%	13.9%	1.0%	75.0%	22.7%	2.3%
Overweight	56.4%	34.1%	9.5%	64.1%	29.8%	6.2%
Obese I	52.5%	35.9%	11.6%	59.3%	31.9%	8.8%
Obese II/III	65.7%	25.4%	8.9%	61.5%	29.8%	8.7%

BCSC, Breast Cancer Surveillance Consortium

^*^Advanced cancer risk is calculated based on a biennial mammography regimen

Mean BCSC 5-year invasive breast cancer risk increased steadily with age in all racial and ethnic groups except for Asian women, among whom risk plateaued at age 60 (Fig. 2B). White women and women reporting other or multiple races had the highest mean invasive cancer risk, with Black women having slightly lower mean risk, and Hispanic and Asian women having markedly lower mean risk scores. The proportion of women with intermediate/high invasive cancer risk was 40.4% for White women, 31.7% for Black women, 31.0% for other/multiple races, 11.4% for Hispanic women, and 6.5% for Asian women (Table 4). The proportion of women with intermediate/high invasive breast cancer risk was higher among women with dense vs. non-dense breasts and among overweight and obese women compared to normal or underweight women.

Among women with intermediate/high advanced cancer risk, 17.5% were age 40–49, 25.7% were Black, 69.7% had dense breasts, and 81.1% were overweight or obese (Table 5). Among women with intermediate/high invasive breast cancer risk, 5.2% were age 40–49, 11.9% were Black, 56.5% had dense breasts, and 70.6% were overweight or obese. For a hypothetical cohort of 100,000 women aged 40–74 years, targeting of interventions based on intermediate/high advanced cancer risk would identify 5650 women aged 40–49; 8287 Black women; 22,495 women with dense breasts; and 26,174 overweight and obese women. In contrast, targeting interventions based on intermediate/high invasive breast cancer risk would reduce these numbers to 1694 women aged 40–49; 3915 Black women; 18,588 women with dense breasts; and 23,217 overweight and obese women. Interventions targeted based on invasive cancer risk would have concomitant increases in counts of older women, White women, women with non-dense breasts, and normal weight women.

**Table 5:** Characteristics of Women Targeted for Supplemental Breast Cancer Screening Strategies Based on Intermediate/high Advanced/invasive Cancer Risk Alone or in Combination with Breast Density, Applied to Hypothetical Cohort of 100,000 US women aged 40–74. Advanced Cancer Risk is Calculated Based on a Biennial Screening Regimen

	US Population aged 40–74*	6-year advanced cancer risk ≥ 0.38%†	5-year invasive cancer risk ≥ 1.67%†	Women with dense breasts†	Dense breasts and 6-year advanced cancer risk ≥ 0.38%†	Dense breasts and 5-year invasive cancer risk ≥ 1.67%†
			N (row percent)			
Total	100,000 (100.0)	32,270 (32.3)	32,900 (32.9)	46,330 (46.3)	22,490 (22.5)	18,590 (18.6)
			N (column percent)			
Age, years						
40–49	29,190 (29.2)	5,650 (17.5)	1,694 (5.2)	18,361 (39.6)	5,382 (23.9)	1,643 (8.8)
50–59	30,800 (30.8)	10,394 (32.2)	7,027 (21.4)	14,186 (30.6)	7,782 (34.6)	5,560 (29.9)
60–69	28,890 (28.9)	12,024 (37.3)	15,799 (48.0)	10,216 (22.1)	6,927 (30.8)	8,126 (43.7)
70–74	11,120 (11.1)	4,202 (13.0)	8,380 (25.5)	3,572 (7.7)	2,397 (10.7)	3,261 (17.5)
Race and ethnicity						
Asian	6,110 (6.1)	887 (2.8)	398 (1.2)	4,045 (8.7)	778 (3.5)	355 (1.9)
Black	12,360 (12.4)	8,287 (25.7)	3,915 (11.9)	5,096 (11.0)	4,345 (19.3)	2,136 (11.5)
Hispanic	14,250 (14.3)	3,346 (10.4)	1,622 (4.9)	6,356 (13.7)	2,793 (12.4)	1,086 (5.8)
White	65,230 (65.2)	19,039 (59.0)	26,330 (80.0)	29,850 (64.4)	14,079 (62.6)	14,643 (78.8)
Other/multiple	2,050 (2.1)	710 (2.2)	635 (1.9)	982 (2.1)	493 (2.2)	370 (2.0)
Breast density						
Almost entirely fat	9,670 (9.7)	19 (0.1)	872 (2.7)	0 (0.0)	0 (0.0)	0 (0.0)
Scattered fibroglandular densities	44,000 (44.0)	9,752 (30.2)	13,443 (40.9)	0 (0.0)	0 (0.0)	0 (0.0)
Heterogeneously dense	38,580 (38.6)	18,581 (57.6)	15,624 (47.5)	38,579 (83.3)	18,574 (82.6)	15,627 (84.1)
Extremely dense	7,750 (7.8)	3,914 (12.1)	2,964 (9.0)	7,751 (16.7)	3,916 (17.4)	2,963 (15.9)
Body mass index						
Underweight	1,360 (1.4)	436 (1.4)	207 (0.6)	1,056 (2.3)	409 (1.8)	191 (1.0)
Normal	37,000 (37.0)	5,663 (17.6)	9,475 (28.8)	23,906 (51.6)	5,382 (23.9)	7,609 (40.9)
Overweight	30,040 (30.0)	13,189 (40.9)	10,850 (33.0)	13,172 (28.4)	10,226 (45.5)	6,187 (33.3)
Obese I	17,540 (17.5)	8,264 (25.6)	7,080 (21.5)	5,402 (11.7)	4,230 (18.8)	2,907 (15.6)
Obese II/III	14,050 (14.1)	4,721 (14.6)	5,287 (16.1)	2,794 (6.0)	2,240 (10.0)	1,695 (9.1)

^*^from the US Census^51^ (age, race/ethnicity) and the Breast Cancer Surveillance Consortium (breast density, body mass index)

^†^Breast Cancer Surveillance Consortium data standardized to the US population based on age and race

Women with dense breasts comprised 46.3% of the study population. Among the full sample, 22.5% had dense breasts and intermediate/high advanced cancer risk; 18.6% of women had dense breasts and intermediate/high invasive breast cancer risk (Table 5). 3.9% had extremely dense breasts and intermediate/high advanced cancer risk; and 3.0% had extremely dense breasts and intermediate/high invasive breast cancer risk (eTable 3).

Similar or more pronounced differences in the prevalence of young women, Black women, women with dense breasts, and overweight/obese women were observed for the advanced cancer risk model vs. the invasive cancer risk model when using high risk as the threshold (eTable 4, eFigure).

## DISCUSSION

Our results demonstrate that while there is moderate correlation between estimated risk scores from the BCSC 6-year advanced cancer risk model and the BCSC 5-year invasive breast cancer risk model, there are substantial differences between the two risk models in characteristics of women identified as having elevated risk. Women with intermediate/high BCSC 6-year advanced cancer risk are more likely to be young, Black, overweight/obese, and have dense breasts compared to women with intermediate/high BCSC 5-year invasive breast cancer risk. These findings illustrate the potential impacts on populations selected for consideration of prevention and screening interventions based on advanced vs. invasive cancer risk.

The goal of the current work was to describe who would be identified for risk-based interventions depending on whether an invasive or advanced cancer risk model was used. The models include a similar set of risk factors, though the invasive cancer model additionally includes age at first birth and family history of breast cancer among both first and second degree relatives (compared to first degree only in the advanced cancer risk model). Differences in the characteristics of women with intermediate/high risk could be partially attributable to these differences in the risk factors included in the models but are likely predominantly due to known differences in the strength of associations for prevalent risk factors for advanced vs. invasive cancer risk. Notably, breast density and body mass index are common risk factors more strongly associated with advanced vs. invasive cancer. Both models include age and race and ethnicity, but with different patterns in their risk associations. Black race is a very strong risk factor for an advanced cancer diagnosis likely because of high incidence of triple negative disease,^31^ whereas Black women on average have moderate invasive cancer risk similar to White women. Our results illustrate how these differences in the underlying risk factor associations for the different outcomes translate to differences in the populations identified as intermediate and high risk.

The invasive and advanced cancer risk models identify similar proportions of the population as having intermediate (25.3% vs. 27.8% for advanced vs. invasive cancer risk) and high (6.6% vs. 4.7% for advanced vs. invasive cancer risk) risk. However, our results demonstrate that the characteristics of these populations identified as having intermediate or high risk differ substantially. Interventions focusing on women with elevated invasive breast cancer risk will identify more women who are older, identify as White, have non-dense breasts, and have a normal body mass index relative to those identified as having elevated advanced cancer risk.

Breast cancer screening guidelines in the USA remain largely age-based,^30^ with the notable exception of a recommendation for supplemental MRI screening among women with high-penetrance genetic mutations or other factors associated with a lifetime invasive breast cancer risk greater than 20%.^32,33^ There is active debate regarding recommendations for age to begin and end mammography screening, mammography screening interval, and appropriate additional populations for supplemental ultrasound or MRI screening—particularly among women with dense breasts.^34^ Researchers have evaluated risk-based screening strategies that could potentially optimize the balance of benefits and harms by directing increased screening intensity to women at higher invasive breast cancer risk.^7,16–20^ The recently developed BCSC advanced cancer risk model enables new strategies targeted to women at elevated advanced cancer risk. An advanced-stage breast cancer diagnosis is associated with elevated mortality risk, whereas an early-stage breast cancer diagnosis has a high survival rate.^22^ Screening mammography reduces breast cancer mortality by reducing the incidence of advanced breast cancer.^35^ Consequently, advanced cancer has emerged as an important intermediate outcome in breast cancer screening trials and observational studies assessing screening intervals and new screening modalities.^36–40^ Targeting enhanced screening strategies based on advanced cancer risk rather than invasive cancer risk could result in a better balance between the benefits and harms of those interventions, though further research is needed to provide direct evidence regarding the comparative effectiveness of such strategies. Our results indicate that targeting women at elevated advanced cancer risk would more than double the representation of Black women and women aged 40–49, compared with strategies relying on elevated invasive cancer risk. There would also be moderate increases in the representation of women aged 50–59, women with dense breasts, and women who are overweight or obese.

A number of organizations recommend consideration of chemoprevention agents for breast cancer risk reduction among women at high invasive breast cancer risk (5-year risk ≥ 3.0%), with some groups also recommending consideration among women with intermediate risk (5-year risk ≥ 1.67%).^15,41–43^ There have also been calls to incorporate the risk of aggressive cancers and advanced cancers into breast cancer chemoprevention strategies,^44^ which currently rely on invasive breast cancer risk.^15,41–43^ Our results can be used to characterize the populations identified based on intermediate vs. high invasive risk, and also demonstrate differences compared to elevated advanced cancer risk.

Limitations of our analyses include the restriction to two BCSC risk prediction models, which were designed for women in the general population and not for women with high-penetrance genetic mutations. The risk models are valid for women with a recent mammogram but otherwise do not consider a woman’s prior screening history. Numerous other invasive breast cancer risk models exist, some of which consider additional risk factors, and findings may differ across models. However, the BCSC invasive risk model was developed in a diverse population,^10,25,26^ has been validated in numerous external populations,^8,45,46^ includes similar risk factors to many other invasive breast cancer prediction models,^47^ and consistently performs well compared to other risk models.^8,48^ Screening outcomes within the BCSC population have been widely reported in prior literature,^23,24^ including studies relating screening outcomes to invasive and advanced cancer risk.^20,40^ We focused on 5-year risk of invasive breast cancer and cumulative 6-year risk of advanced cancer and did not evaluate models for lifetime risk of breast cancer. On average, lifetime risk declines with age while short-term breast cancer risk increases with age.^49^ Thus, populations selected based on high lifetime risk will tend to be younger women.^50^ Our study does not estimate outcomes associated with any risk-based strategies or establish which model is most appropriate for certain risk-based interventions; further research is needed to evaluate the comparative effectiveness of breast cancer screening and prevention strategies that rely on advanced vs. invasive cancer risk.

In summary, our results indicate substantial differences in sociodemographic and risk factor prevalence among women with elevated BCSC 5-year invasive vs. 6-year advanced breast cancer risk. The advanced cancer and invasive cancer risk models identify different groups of women as elevated risk. Researchers, clinicians, and policymakers should be cognizant of these differences and their potential impacts on the effectiveness of risk-based breast cancer screening and primary prevention strategies.

## Supplementary Information

Below is the link to the electronic supplementary material.

Supplementary file1 (DOCX 434 KB)

## References

[1] **Shieh Y, Eklund M, Madlensky L, et al.** Breast cancer screening in the precision medicine Era: risk-based screening in a population-based Trial. J Natl Cancer Inst. 2017;109(5). 10.1093/jnci/djw290. 28130475

[2] **Yala A, Mikhael PG, Lehman C.** Optimizing risk-based breast cancer screening policies with reinforcement learning. Nat Med. 2022;28(1):136-143. 10.1038/s41591-021-01599-w. 35027757

[3] **Mukama T, Kharazmi E, Xu X.** Risk-Adapted Starting Age of Screening for Relatives of Patients With Breast Cancer. JAMA Oncol. 2020;6(1):68-74. 10.1001/jamaoncol.2019.3876. PMC6865319 31725845

[4] **Moorthie S, Babb de Villiers C, Burton H, et al.** Towards implementation of comprehensive breast cancer risk prediction tools in health care for personalised prevention. Prev Med. 2022;159:107075. 10.1016/j.ypmed.2022.107075. 35526672

[5] **Pashayan N, Antoniou AC, Ivanus U.** Personalized early detection and prevention of breast cancer: ENVISION consensus statement. Nat Rev Clin Oncol. 2020;17(11):687-705. 10.1038/s41571-020-0388-9. PMC7567644 32555420

[6] **Pashayan N, Morris S, Gilbert FJ, Pharoah PD.** Cost-effectiveness and Benefit-to-Harm Ratio of Risk-Stratified Screening for Breast Cancer: A Life-Table Model. JAMA Oncol. 2018;4(11):1504-1510. 10.1001/jamaoncol.2018.1901. PMC6230256 29978189

[7] **van Den Broek JJ, Schechter CB, van Ravesteyn NT.** Personalizing Breast Cancer Screening Based on Polygenic Risk and Family History. J Natl Cancer Inst. 2021;113(4):434-442. 10.1093/jnci/djaa127. PMC8599807 32853342

[8] **McCarthy AM, Guan Z, Welch M.** Performance of Breast Cancer Risk-Assessment Models in a Large Mammography Cohort. J Natl Cancer Inst. 2020;112(5):489-497. 10.1093/jnci/djz177. PMC7225681 31556450

[9] **Louro J, Posso M, Hilton Boon M.** A systematic review and quality assessment of individualised breast cancer risk prediction models. Br J Cancer. 2019;121(1):76-85. 10.1038/s41416-019-0476-8. PMC6738106 31114019

[10] **Gard CC, Tice JA, Miglioretti DL.** Extending the Breast Cancer Surveillance Consortium Model of Invasive Breast Cancer. J Clin Oncol. 2024;42(7):779-789. 10.1200/JCO.22.02470. PMC10906584 37976443

[11] **Tice JA, Cummings SR, Smith-Bindman R, Ichikawa L, Barlow WE, Kerlikowske K.** Using clinical factors and mammographic breast density to estimate breast cancer risk: development and validation of a new predictive model. Ann Intern Med. 2008;148(5):337-347. 148/5/337[pii]. 10.7326/0003-4819-148-5-200803040-00004 PMC2674327 18316752

[12] **Gail MH, Brinton LA, Byar DP**. Projecting individualized probabilities of developing breast cancer for white females who are being examined annually. JNCI: J Nat Cancer Instit. 1989;81(24):1879-86. 10.1093/jnci/81.24.1879 2593165

[13] **Costantino JP, Gail MH, Pee D.** Validation studies for models projecting the risk of invasive and total breast cancer incidence. J Natl Cancer Inst. 1999;91(18):1541-1548. 10.1093/jnci/91.18.1541. 10491430

[14] **Tyrer J, Duffy SW, Cuzick J.** A breast cancer prediction model incorporating familial and personal risk factors. Stat Med. 2004;23(7):1111-1130. 10.1002/sim.1668. 15057881

[15] U. S. Preventive Services Task Force, **Owens DK, Davidson KW, et al.** Medication Use to Reduce Risk of Breast Cancer: US Preventive Services Task Force Recommendation Statement. JAMA. 2019;322(9):857–867. 10.1001/jama.2019.11885. 31479144

[16] **Van Ravesteyn NT, Miglioretti DL, Stout NK.** Tipping the balance of benefits and harms to favor screening mammography starting at age 40 years: a comparative modeling study of risk. Ann Int Med. 2012;156(9):609-17. 10.7326/0003-4819-156-9-201205010-00002. PMC3520058 22547470

[17] **Trentham-Dietz A, Kerlikowske K, Stout NK.** Tailoring Breast Cancer Screening Intervals by Breast Density and Risk for Women Aged 50 Years or Older: Collaborative Modeling of Screening Outcomes. Ann Intern Med. 2016;165(10):700-712. 10.7326/M16-0476. PMC5125086 27548583

[18] **Kerlikowske K, Sprague BL, Tosteson AN.** Strategies to Identify Women at High Risk of Advanced Breast Cancer During Routine Screening for Discussion of Supplemental Imaging. JAMA Intern Med. 2019;179(9):1230-1239. 10.1001/jamainternmed.2019.1758. PMC6604099 31260054

[19] **Kerlikowske K, Zhu W, Tosteson AN.** Breast Cancer Surveillance Consortium*. Identifying women with dense breasts at high risk for interval cancer: a cohort study. Ann Int Med. 2015;162(10):673-81. https://doi.org/10.7326/M14-1465. 10.7326/M14-1465 PMC4443857 25984843

[20] **Sprague BL, Ichikawa L, Eavey J.** Performance of Supplemental US Screening in Women with Dense Breasts and Varying Breast Cancer Risk: Results from the Breast Cancer Surveillance Consortium. Radiology. 2024;312(2):e232380. 10.1148/radiol.232380. PMC11366666 39105648

[21] **Howlader N, Cronin KA, Kurian AW, Andridge R.** Differences in Breast Cancer Survival by Molecular Subtypes in the United States. Cancer Epidemiol Biomarkers Prev. 2018;27(6):619-626. 10.1158/1055-9965.EPI-17-0627. 29593010

[22] **Kerlikowske K, Bissell MC, Sprague BL.** Advanced Breast Cancer Definitions by Staging System Examined in the Breast Cancer Surveillance Consortium. J Natl Cancer Inst. 2021;113(7): 909-916. 10.1093/jnci/djaa176. PMC8491791 33169794

[23] **Kerlikowske K, Chen S, Golmakani MK.** Cumulative Advanced Breast Cancer Risk Prediction Model Developed in a Screening Mammography Population. J Natl Cancer Inst. 2022;114(5):676-685. 10.1093/jnci/djac008. PMC9086807 35026019

[24] **Lee CI, Abraham L, Miglioretti DL.** National Performance Benchmarks for Screening Digital Breast Tomosynthesis: Update from the Breast Cancer Surveillance Consortium. Radiology. 2023;307(4):e222499. 10.1148/radiol.222499. PMC10323294 37039687

[25] **Lee CI, Bogart A, Germino JC.** Availability of Advanced Breast Imaging at Screening Facilities Serving Vulnerable Populations. J Med Screen. 2016;23(1):24-30. 10.1177/0969141315591616. PMC4679713 26078275

[26] **Lee CI, Zhu W, Onega T, et al.** Comparative access to and use of digital breast tomosynthesis screening by Women's Race/Ethnicity and Socioeconomic Status. JAMA Netw Open. 2021;4(2):e2037546. 10.1001/jamanetworkopen.2020.37546. PMC7896194 33606032

[27] American College of Radiology. ACR BI-RADS^®^ - Mammography. 5th Edition. ACR BI-RADS Atlas: Breast Imaging Reporting and Data System. American College of Radiology; 2013.

[28] **Sprague BL, Kerlikowske K, Bowles EJ.** Trends in Clinical Breast Density Assessment From the Breast Cancer Surveillance Consortium. J Natl Cancer Inst. 2019;111(6):629-632. 10.1093/jnci/djy210. PMC6579740 30624682

[29] **Tice JA, Miglioretti DL, Li CS, Vachon CM, Gard CC, Kerlikowske K.** Breast Density and Benign Breast Disease: Risk Assessment to Identify Women at High Risk of Breast Cancer. J Clin Oncol. 2015;33(28):3137-3143. 10.1200/JCO.2015.60.8869. PMC4582144 26282663

[30] U. S. Preventive Services Task Force, **Nicholson WK, Silverstein M, et al.** Screening for Breast Cancer: US Preventive Services Task Force Recommendation Statement. JAMA. 2024. 10.1001/jama.2024.5534.

[31] **Li NHY, Li CI. **Incidence Rate Trends of Breast Cancer Overall and by Molecular Subtype by Race and Ethnicity and Age. JAMA Netw Open. 2025;8(1):e2456142. 10.1001/jamanetworkopen.2024.56142. PMC11762241 39853979

[32] ** Smith RA, Andrews KS, Brooks D.** Cancer screening in the United States, 2017: A review of current American Cancer Society guidelines and current issues in cancer screening. CA Cancer J Clin. 2017;67(2):100-121. 10.3322/caac.21392. 28170086

[33] **Expert Panel on Breast Imaging, Niell BL, Jochelson MS, et al.** ACR Appropriateness Criteria(R) Female Breast Cancer Screening: 2023 Update. J Am Coll Radiol. 2024;21(6S):S126-S143. 10.1016/j.jacr.2024.02.019. 38823941

[34] **Expert Panel on Breast Imaging, Weinstein SP, Slanetz PJ, et al. **ACR Appropriateness Criteria(R) supplemental breast cancer screening based on breast density. J Am Coll Radiol. 2021;18(11S):S456-S473. 10.1016/j.jacr.2021.09.002. 34794600

[35] **Autier P, Héry C, Haukka J, Boniol M, Byrnes G.** Advanced breast cancer and breast cancer mortality in randomized controlled trials on mammography screening. J Clin Oncol. 2009;27(35):5919-5923. 10.1200/JCO.2009.22.7041. 19884547

[36] **Hu P, Steingrimsson JA, Cole E.** Design considerations and challenges in the CHinA National CancEr Screening (CHANCES) trial and Tomosynthesis Mammographic Imaging Screening Trial (TMIST). J Natl Cancer Inst Monogr. 2025;68:42-48. 10.1093/jncimonographs/lgae049. PMC11848039 39989037

[37] **Feng X, Zahed H, Onwuka J.** Cancer Stage Compared With Mortality as End Points in Randomized Clinical Trials of Cancer Screening: A Systematic Review and Meta-Analysis. JAMA. 2024;331(22):1910-1917. 10.1001/jama.2024.5814. PMC11000135 38583868

[38] **Miglioretti DL, Zhu W, Kerlikowske K.** Breast cancer surveillance consortium. breast tumor prognostic characteristics and biennial vs annual mammography, age, and menopausal status. JAMA Oncol. 2015;1(8):1069-77. 10.1001/jamaoncol.2015.3084. PMC4644100 26501844

[39] **Kerlikowske K, Zhu W, Su YR.** Supplemental magnetic resonance imaging plus mammography compared with magnetic resonance imaging or mammography by extent of breast density. J Natl Cancer Inst. 2024;116(2):249-257. 10.1093/jnci/djad201. PMC10852604 37897090

[40] **Kerlikowske K, Su YR, Sprague BL.** Association of screening with digital breast tomosynthesis vs digital mammography with risk of interval invasive and advanced breast cancer. Jama. 2022;327(22):2220-30. 10.1001/jama.2022.7672. PMC9198754 35699706

[41] **Visvanathan K, Fabian CJ, Bantug E.** Use of Endocrine Therapy for Breast Cancer Risk Reduction: ASCO Clinical Practice Guideline Update. J Clin Oncol. 2019;37(33):3152-3165. 10.1200/JCO.19.01472. 31479306

[42] **Bevers TB, Ward JH, Arun BK, et al.** Breast Cancer Risk Reduction, Version 2.2015. J Natl Compr Canc Netw. 2015;13(7):880–915. 10.6004/jnccn.2015.0105. 26150582

[43] **Farkas A, Vanderberg R, Merriam S, DiNardo D.** Breast Cancer Chemoprevention: A Practical Guide for the Primary Care Provider. J Womens Health (Larchmt). 2020;29(1):46-56. 10.1089/jwh.2018.7643. 31560601

[44] **Chlebowski RT, Aragaki AK, Pan K.** Breast Cancer Prevention: Time for Change. JCO Oncol Pract. 2021;17(12):709-716 10.1200/OP.21.00343. PMC8677965 34319769

[45] **Tice JA, Bissell MC, Miglioretti DL. ** Validation of the breast cancer surveillance consortium model of breast cancer risk. Breast Cancer Res Treat. 2019;175(2)5:19-523. 10.1007/s10549-019-05167-2. PMC7138025 30796654

[46] **Vachon CM, Pankratz VS, Scott CG, et al.** The contributions of breast density and common genetic variation to breast cancer risk. J Natl Cancer Inst. 2015;107(5):dju397. 10.1093/jnci/dju397. PMC4598340 25745020

[47] **Kim G, Bahl M.** Assessing Risk of Breast Cancer: A Review of Risk Prediction Models J Breast Imaging. 2021;3(2):144-155. 10.1093/jbi/wbab001. PMC7980704 33778488

[48] **McCarthy AM, Liu Y, Ehsan S, et al. **Validation of Breast Cancer Risk Models by Race/Ethnicity, Family History and Molecular Subtypes. Cancers (Basel). 2021;14(1). 10.3390/cancers14010045. PMC8750569 35008209

[49] **Coopey SB, Acar A, Griffin M, Cintolo-Gonzalez J, Semine A, Hughes KS.** The impact of patient age on breast cancer risk prediction models. Breast J. 2018;24(4):592-598. 10.1111/tbj.12976. 29316072

[50] **Hollingsworth AB, Li FY, Morse AN.** Lifetime risks for breast cancer are age-discriminatory when used for high-risk screening with MRI. Cancer epidemiology. Jun 2022;78:102122. 10.1016/j.canep.2022.102122. 35279431

[51] United States Department of Commerce, U.S. Census Bureau, Population Division. Single-race Population Estimates, United States, 2020–2022, on CDC WONDER Online Database. http://wonder.cdc.gov/single-race-single-year-v2022.html . Accessed 6 Dec 2024.

