# Supplementary material for: Advanced Versus Invasive Breast Cancer Risk in a Screening Population: Implications for Risk-based Prevention and Screening Strategies
Source: J Gen Intern Med. 2026 Feb 18;41(12):3329–38. doi: 10.1007/s11606-026-10237-5 (PMC13081705; doi:10.1007/s11606-026-10237-5)
Supplement: Supplementary file 1 [file 11606_2026_10237_MOESM1_ESM.docx]

**Appendix: Supplemental Tables and Figures**

**eTable 1.** Cross-classification of BCSC 6-year advanced breast cancer risk and BCSC 5-year invasive breast cancer risk among women with dense breasts.

**eTable 2.** Cross-classification of BCSC 6-year advanced breast cancer risk and BCSC 5-year invasive breast cancer risk among women with non-dense breasts.

**eTable 3**. Characteristics of women targeted for supplemental breast cancer screening strategies based on having intermediate/high advanced/invasive cancer risk and extremely dense breasts, applied to hypothetical cohort of 100,000 US women aged 40-74. Advanced cancer risk is calculated based on a biennial screening regimen.

**eTable 4**. Characteristics of women targeted for supplemental breast cancer screening strategies based on high advanced/invasive cancer risk alone or in combination with breast density, applied to hypothetical cohort of 100,000 US women aged 40-74. Advanced cancer risk is calculated based on a biennial screening regimen.

**eFigure.** Race and ethnicity distribution of women targeted for supplemental breast cancer screening strategies based on elevated advanced or invasive cancer risk, applied to a hypothetical cohort of 100,000 US women aged 40-74. Advanced cancer risk is calculated based on a biennial screening regimen.

**eTable 1.** Cross-classification of BCSC 6-year advanced breast cancer risk and BCSC 5-year invasive breast cancer risk among women with dense breasts.

|  | BCSC 6-year advanced breast cancer risk* | | |
| --- | --- | --- | --- |
| BCSC 5-year invasive breast cancer risk | Low/Average | Intermediate | High |
|  | (<0.38%) | (0.38-0.66%) | (>0.66%) |
| Low/Average (<1.67%) | 161,115 (44.6%) | 54,793 (15.2%) | 3,560 (1.0%) |
| Intermediate (1.67 - <3.00%) | 29,503 (8.2%) | 62,392 (17.3%) | 24,002 (6.7%) |
| High (≥3.00%) | 893 (0.3%) | 10,229 (2.8%) | 14,574 (4.0%) |

BCSC, Breast Cancer Surveillance Consortium

*Advanced cancer risk is calculated based on a biennial mammography regimen.

**eTable 2.** Cross-classification of BCSC 6-year advanced breast cancer risk and BCSC 5-year invasive breast cancer risk among women with non-dense breasts.

|  | BCSC 6-year advanced breast cancer risk* | | |
| --- | --- | --- | --- |
| BCSC 5-year invasive breast cancer risk | Low/Average | Intermediate | High |
|  | (<0.38%) | (0.38-0.66%) | (>0.66%) |
| Low/Average (<1.67%) | 268,417 (67.8%) | 22,967 (5.8%) | 415 (0.1%) |
| Intermediate (1.67 - <3.00%) | 54,009 (13.6%) | 34,717 (8.8%) | 5,632 (1.4%) |
| High (≥3.00%) | 1,274 (0.3%) | 6,490 (1.6%) | 1,989 (0.5%) |

BCSC, Breast Cancer Surveillance Consortium

*Advanced cancer risk is calculated based on a biennial mammography regimen.

**eTable 3**. Characteristics of women targeted for supplemental breast cancer screening strategies based on having intermediate/high advanced/invasive cancer risk and extremely dense breasts, applied to hypothetical cohort of 100,000 US women aged 40-74. Advanced cancer risk is calculated based on a biennial screening regimen.

|  | | US Population  aged 40-74* |  | Extremely dense breasts and 6-year advanced cancer risk≥0.38%† | Extremely dense breasts and 5-year invasive cancer risk≥1.67%† |
| --- | --- | --- | --- | --- | --- |
|  | N (row percent) | | | | |
| Total | | 100,000 (100.0) |  | 3,910 (3.9) | 2,960 (3.0) |
|  | |  |  |  |  |
|  | N (column percent) | | | | |
| Age, years | |  |  |  |  |
| 40-49 | | 29,190 (29.2) |  | 1,315 (33.6) | 668 (22.6) |
| 50-59 | | 30,800 (30.8) |  | 1,299 (33.2) | 1,065 (36.0) |
| 60-69 | | 28,890 (28.9) |  | 1,018 (26.0) | 937 (31.7) |
| 70-74 | | 11,120 (11.1) |  | 279 (7.1) | 289 (9.8) |
|  | |  |  |  |  |
| Race and ethnicity | |  |  |  |  |
| Asian | | 6,110 (6.1) |  | 251 (6.4) | 110 (3.7) |
| Black | | 12,360 (12.4) |  | 475 (12.2) | 206 (7.0) |
| Hispanic | | 14,250 (14.3) |  | 443 (11.3) | 160 (5.4) |
| White | | 65,230 (65.2) |  | 2,655 (67.9) | 2,420 (81.7) |
| Other/multiple | | 2,050 (2.1) |  | 86 (2.2) | 64 (2.2) |
|  | |  |  |  |  |
| Breast density | |  |  |  |  |
| Almost entirely fat | | 9,670 (9.7) |  | 0 (0.0) | 0 (0.0) |
| Scattered fibroglandular densities | | 44,000 (44.0) |  | 0 (0.0) | 0 (0.0) |
| Heterogeneously dense | | 38,580 (38.6) |  | 0 (0.0) | 0 (0.0) |
| Extremely dense | | 7,750 (7.8) |  | 3,910 (100.0) | 2,960 (100.0) |
|  | |  |  |  |  |
| Body mass index | |  |  |  |  |
| Underweight | | 1,360 (1.4) |  | 215 (5.5) | 91 (3.1) |
| Normal | | 37,000 (37.0) |  | 1,964 (50.2) | 1,872 (63.2) |
| Overweight | | 30,040 (30.0) |  | 1,299 (33.2) | 700 (23.6) |
| Obese I | | 17,540 (17.5) |  | 316 (8.1) | 208 (7.0) |
| Obese II/III | | 14,050 (14.1) |  | 116 (3.0) | 90 (3.0) |

*from the US Census^52^ (age, race/ethnicity) and the Breast Cancer Surveillance Consortium (breast density, body mass index).

†Breast Cancer Surveillance Consortium data standardized to the US population based on age and race.

**eTable 4**. Characteristics of women targeted for supplemental breast cancer screening strategies based on high advanced/invasive cancer risk alone or in combination with breast density, applied to hypothetical cohort of 100,000 US women aged 40-74. Advanced cancer risk is calculated based on a biennial screening regimen.

|  | US Population aged 40-74* |  | 6-year advanced cancer risk >0.66%† | 5-year invasive breast cancer risk ≥3.00%† |  | Women with dense breasts† |  | Dense breasts and 6-year advanced cancer risk>0.66%† | Dense breasts and 5-year invasive cancer risk≥3.00%† |  | Extremely dense breasts and 6-year advanced cancer risk>0.66%† | Extremely dense breasts and 5-year invasive cancer risk≥3.00%† |
| --- | --- | --- | --- | --- | --- | --- | --- | --- | --- | --- | --- | --- |
|  | N (row percent) | | | | | | | | | | | |
| Total | 100,000 (100.0) |  | 6,690 (6.7) | 4,810 (4.8) |  | 46,330 (46.3) |  | 5,630 (5.6) | 3,470 (3.5) |  | 960 (1.0) | 480 (0.5) |
|  |  |  |  |  |  |  |  |  |  |  |  |  |
|  | N (column percent) | | | | | | | | | | | |
| Age, years |  |  |  |  |  |  |  |  |  |  |  |  |
| 40-49 | 29,190 (29.2) |  | 767 (11.5) | 85 (1.8) |  | 18,361 (39.6) |  | 759 (13.5) | 84 (2.4) |  | 218 (22.7) | 47 (9.7) |
| 50-59 | 30,800 (30.8) |  | 1,991 (29.8) | 626 (13.0) |  | 14,186 (30.6) |  | 1,798 (31.9) | 568 (16.4) |  | 377 (39.3) | 138 (28.8) |
| 60-69 | 28,890 (28.9) |  | 2,922 (43.7) | 2,438 (50.7) |  | 10,216 (22.1) |  | 2,271 (40.3) | 1,798 (51.8) |  | 286 (29.8) | 214 (44.5) |
| 70-74 | 11,120 (11.1) |  | 1,010 (15.1) | 1,662 (34.6) |  | 3,572 (7.7) |  | 802 (14.3) | 1,020 (29.4) |  | 79 (8.2) | 82 (17.0) |
|  |  |  |  |  |  |  |  |  |  |  |  |  |
| Race and ethnicity |  |  |  |  |  |  |  |  |  |  |  |  |
| Asian | 6,110 (6.1) |  | 74 (1.1) | 17 (0.4) |  | 4,045 (8.7) |  | 73 (1.3) | 16 (0.5) |  | 26 (2.7) | 5 (1.1) |
| Black | 12,360 (12.4) |  | 3,036 (45.4) | 378 (7.9) |  | 5,096 (11.0) |  | 2,192 (38.9) | 285 (8.2) |  | 234 (24.4) | 26 (5.5) |
| Hispanic | 14,250 (14.3) |  | 473 (7.1) | 95 (2.0) |  | 6,356 (13.7) |  | 462 (8.2) | 83 (2.4) |  | 101 (10.5) | 13 (2.7) |
| White | 65,230 (65.2) |  | 2,981 (44.6) | 4,228 (87.9) |  | 29,850 (64.4) |  | 2,792 (49.6) | 3,021 (87.1) |  | 576 (60.0) | 424 (88.4) |
| Other/multiple | 2,050 (2.1) |  | 126 (1.9) | 92 (1.9) |  | 982 (2.1) |  | 111 (2.0) | 66 (1.9) |  | 23 (2.4) | 11 (2.3) |
|  |  |  |  |  |  |  |  |  |  |  |  |  |
| Breast density |  |  |  |  |  |  |  |  |  |  |  |  |
| Almost entirely fat | 9,670 (9.7) |  | 0 (0.0) | 25 (0.5) |  | 0 (0.0) |  | 0 (0.0) | 0 (0.0) |  | 0 (0.0) | 0 (0.0) |
| Scattered fibroglandular densities | 44,000 (44.0) |  | 1,057 (15.8) | 1,319 (27.4) |  | 0 (0.0) |  | 0 (0.0) | 0 (0.0) |  | 0 (0.0) | 0 (0.0) |
| Heterogeneously dense | 38,580 (38.6) |  | 4,672 (69.8) | 2,983 (62.0) |  | 38,579 (83.3) |  | 4,669 (82.9) | 2,987 (86.1) |  | 0 (0.0) | 0 (0.0) |
| Extremely dense | 7,750 (7.8) |  | 961 (14.4) | 483 (10.0) |  | 7,751 (16.7) |  | 961 (17.1) | 483 (13.9) |  | 960 (100.0) | 480 (100.0) |
|  |  |  |  |  |  |  |  |  |  |  |  |  |
| Body mass index |  |  |  |  |  |  |  |  |  |  |  |  |
| Underweight | 1,360 (1.4) |  | 68 (1.0) | 6 (0.1) |  | 1,056 (2.3) |  | 67 (1.2) | 7 (0.2) |  | 42 (4.4) | 4 (0.8) |
| Normal | 37,000 (37.0) |  | 400 (6.0) | 762 (15.9) |  | 23,906 (51.6) |  | 392 (7.0) | 719 (20.7) |  | 209 (21.8) | 215 (44.8) |
| Overweight | 30,040 (30.0) |  | 2,922 (43.7) | 1,636 (34.0) |  | 13,172 (28.4) |  | 2,708 (48.1) | 1,319 (38.0) |  | 482 (50.2) | 162 (33.8) |
| Obese I | 17,540 (17.5) |  | 2,050 (30.7) | 1,352 (28.1) |  | 5,402 (11.7) |  | 1,588 (28.2) | 866 (25.0) |  | 164 (17.1) | 69 (14.3) |
| Obese II/III | 14,050 (14.1) |  | 1,249 (18.7) | 1,054 (21.9) |  | 2,794 (6.0) |  | 874 (15.5) | 560 (16.1) |  | 63 (6.5) | 30 (6.3) |

*from the US Census^52^ (age, race/ethnicity) and the Breast Cancer Surveillance Consortium (breast density, body mass index).

†Breast Cancer Surveillance Consortium data standardized to the US population based on age and race.

**eFigure.** Race and ethnicity distribution of women targeted for supplemental breast cancer screening strategies based on elevated advanced or invasive cancer risk, applied to a hypothetical cohort of 100,000 US women aged 40-74. Advanced cancer risk is calculated based on a biennial screening regimen.
